# From lifespan to healthspan: the role of nutrition in healthy ageing

**DOI:** 10.1017/jns.2020.26

**Published:** 2020-08-24

**Authors:** Kremlin Wickramasinghe, John C. Mathers, Suzan Wopereis, Daniel S. Marsman, James C. Griffiths

**Affiliations:** 1 WHO European Office for Prevention and Control of Noncommunicable Diseases (NCD Office), Moscow, Russian Federation; 2 Human Nutrition Research Centre, Population Health Sciences Institute, Newcastle University, Newcastle upon Tyne NE2 4HH, UK; 3 Research Group Microbiology and Systems Biology, Netherlands Organization for Applied Scientific Research (TNO), Zeist, NL-3704 HE, The Netherlands; 4 Global Product Stewardship, P&G Health, Mason, OH 45040, USA; 5 International and Scientific Affairs, Council for Responsible Nutrition-International, Washington, DC 20036, USA

**Keywords:** Ageing, Healthspan, Lifespan, Longevity, Non-communicable diseases, Nutrition, Phenotypic flexibility

## Abstract

Across the globe, there has been a marked increase in longevity, but significant inequalities remain. These are exacerbated by inadequate access to proper nutrition and health care services and to reliable information to make the decisions related to nutrition and health care. Many in economically developing as well as developed societies are plagued with the double-burden of energy excess and undernutrition. This has resulted in mental and physical deterioration, increased non-communicable disease rates, lost productivity, increased medical costs and reduced quality of life. While adequate nutrition is fundamental to good health at all stages of the life course, the impact of diet on prolonging good quality of life during ageing remains unclear. For progress to continue, there is need for new and/or innovative approaches to promoting health as individuals age, as well as qualitative and quantitative biomarkers and other accepted tools that can measure improvements in physiological integrity throughout life. A framework for progress has been proposed by the World Health Organization in their Global Strategy and Action Plan on Ageing and Health. Here, we focused on the impact of nutrition within this framework, which takes a broad, person-centred emphasis on healthy ageing, stressing the need to better understand each individual's intrinsic capacity, their functional abilities at various life stages, and the impact of their mental, and physical health, as well as the environments they inhabit.

The human lifespan has substantially extended since the 1900s, due largely to interventions that have reduced infant and childhood mortality, coupled with medical-surgical advances that have had a particular impact on older people. However, this lifespan expansion has not resulted in robust health for everyone during ageing and there has been a substantial increase in age-associated morbidity. The research field of healthy ageing has developed to identify risk factors impacting on health and quality of life and to provide evidence of effective and acceptable interventions.

The concept of a well-balanced diet has long been advocated for a healthy life. However, more recently it has become apparent that nutritional needs vary greatly across and within age groups, and thus, generic dietary recommendations may not be optimal for everyone in the population. This inter-individual variability in nutritional needs is likely to be exacerbated with increasing chronological age, due to the effects of environmental stressors, lifestyle choices and chronic disease. Adequate physical activity, optimal nutrition, restorative sleep, and the minimisation of personal risk factors are all critical to a healthy lifespan. The WHO report on healthy ageing^(1,2)^ notes that within intrinsic capacity, an individual has countless choices that lead to good health (or lack thereof) both now and later in life^(3–5)^.

A long-term view of healthy ageing requires respect for the highly diverse needs and expectations across the lifespan. In the present paper, we explored diverse lifestyle and risk factors impacting healthy ageing, and the overarching role of good nutrition in meeting the needs of heterogeneous older populations. While medical-surgical interventions and dietetic practices have always been patient-focused individual matters, more recent research has focused on societal dietary advice to improve health and well-being. Understanding individual extrinsic and intrinsic influencers (including individual behaviours and individual-specific data) may provide a basis for personalised nutritional interventions to enhance human healthspan.

## The public health significance of improving healthspan (*v*. lifespan)

For more than 150 years, life expectancy has been increasing due, initially, to reductions in childhood mortality and, since the middle of the 20th century, to declining late-life mortality^(6)^. This has led to accelerating growth in the numbers of centenarians in countries with the greatest life expectancy^(7)^.

But not for everyone, everywhere. Inequalities abound; for example, life expectancy in Europe varies markedly between Eastern European and Western European countries. Besides geography, gender has a great impact on humans’ life expectancy. Even though men self-report a higher subjective health than women, their life expectancy can be up to 10 years shorter, perhaps due to a lifestyle, fraught with more risk factors which lead to higher rates of high blood pressure, high blood sugar and cardiovascular disease (CVD) compared to women^(8)^.

In Eastern European countries, 37 % of male deaths related to non-communicable diseases (NCDs) occur before the age of 60, often caused by cancer^(9)^, exacerbated by men's lower usage of prevention and health care services and the tendency to seek medical help later as compared to women^(10)^. The main NCD risk factors are dietary risks, tobacco, raised systolic blood pressure (BP), alcohol and drug use^(9)^ and may also be due to cultural gender roles and socio-economic factors that affect men more than women^(10)^.

Age-specific prevalence of dementia is falling in some countries, including the UK^(11)^, probably because of reduced smoking, more effective treatment of CVD risk factors, such as raised BP and raised blood lipids and better education. However, even with some positive trends, the growing number of older people means that by 2050, the total number of those living with dementia is expected to be over 130 million (about three times greater than now).

The extension in life expectancy that is resulting from advances in prevention and treatment of multiple diseases is changing the age distribution of the population, which is becoming older. In the year 2015, more than 25 % of the European population was 60+ years old, and by the year 2050, more than 30 % of Europeans will be over 60 years old^(1)^. Such demographic changes demand more knowledge on the unique physiology of elderly subjects. Apart from obvious changes such as in reproductive function and body composition, the ageing process is associated with the metabolic alterations leading to the development of NCDs, such as Diabetes Mellitus type II^(12)^. Further, the accumulation of molecular damage caused by inflammation, oxidation and dysmetabolism^(13–15)^ can lead to the development of CVD, leading causes of mortality and morbidity^(16)^. Therefore, the regulation of inflammation, oxidation and metabolism is paramount to ‘optimal resilient ageing’, i.e., the natural ageing process with the highest possible physical, mental and social well-being, and with the lowest incidence of cardiometabolic diseases^(17)^.

Billions of people around the world are affected by NCDs at all stages of the 'life course', from childhood to old age, with premature death mainly caused by the following four NCDs: cardiovascular diseases; cancers; chronic respiratory diseases; diabetes. These four diseases are largely preventable through public policies that tackle the four main risk factors: tobacco use; harmful use of alcohol; unhealthy diets; physical inactivity^(18)^.

Optimal nutrition and physical activity through the life course are essential for health and quality of life. A life course approach is key to the prevention and control of diet-related NCDs and malnutrition in all its forms. The approach starts by addressing maternal nutritional status and health before and during pregnancy and continues with proper infant feeding practices, including the promotion of exclusive breastfeeding and healthy complementary feeding. Action to encourage healthy diets for children, adolescents and young people is reinforced and sustained by the promotion of a healthy diet during the working life, nutrition for healthy ageing and nutritional care for elderly people. It also includes nutritional care for patients with disease-related nutritional problems, including micronutrient deficiencies^(19)^.

At this time of encouraging health promotion, it is concerning to note that the WHO European Region has the lowest global breastfeeding rates. Social marginalisation, policies in the workplace and the employment market, marketing of breast-milk substitutes, and commercial ‘follow-on’ and complementary foods are just some of the reasons for low breastfeeding rates in this region. Mothers with low socio-economic status, often without employment policies that allow time at home with the newborn, are up to ten times less likely to begin breastfeeding, and this tendency is transmitted through generations^(20)^.

Good nutrition and a healthy diet during pregnancy are critical for a mother's health, as well as that of her child. A healthy diet contains adequate energy, protein, vitamins and minerals, obtained from a variety of foods, including green and orange vegetables, meat, fish, beans, nuts, whole grains and fruit^(21)^. Evidence suggests that proactive nutrition education and counselling may reduce gestational diabetes and support optimal gestational weight gain, reduce the risk of anaemia in late pregnancy and lower the risk of preterm delivery. This strategy aims to increase the diversity and amount of foods consumed; promote adequate weight gain through sufficient and balanced protein and energy intake; and promote consistent and continued use of micronutrient supplements, food supplements or fortified foods^(21)^.

Pregnant women require additional iron and folic acid to meet their own nutritional needs, as well as those of the developing foetus. Evidence has shown that the use of iron and folic acid supplements is associated with a reduced risk of iron deficiency and anaemia in pregnant women and promotes healthy fetal development^(21)^. Breastfeeding has been identified as a protective factor against obesity^(22)^. Therefore, all mothers should be supported to initiate breastfeeding as soon as possible after delivery, ideally within the first hour after the birth of their infant^(21)^.

Good nutrition in infancy and early childhood is the key to ensure optimal child growth and development, as well as better health outcomes later in life, such as the prevention of malnutrition in all its forms, including overweight, obesity and diet-related NCDs^(23)^. A study of the availability, composition and marketing of baby foods in four countries (Austria, Bulgaria, Hungary and Israel) found evidence of widespread inappropriate promotion of commercial foods for infants and young children^(24)^. Despite globally agreed regulations on the promotion of foods for infants and children, many companies that make and sell commercial baby foods fail to comply with these rules. Action is, therefore, required by countries, with the support of the WHO Regional Office, to fully implement this nearly 40-year-old Code of Practice and additional WHO Guidance, in order to prevent the promotion of nutritionally inappropriate products and/or use of inappropriate promotional techniques^(23)^.

It is particularly important to address the issue of high total sugars, use of sweet ingredients and misleading product names. Sugar, fruit juice, concentrated fruit juice and other sweetening agents should not be added to foods for infants and young children. Manufacturers and retailers should comply with the International Code and the WHO Guidance^(23)^.

Promoting sound nutrition and physical activity are essential for the success of the Sustainable Development Goals, the WHO blueprint to achieve a better and more sustainable future for all. In November 2019, the WHO European Office hosted an expert meeting to assemble all stakeholders, from Health Ministers to nutritionists, from academic researchers to culinary professionals, to create effective, functional and, most importantly, reliable action steps. The outcome of this meeting was a framework for all European Member States to adjust national guidelines to take a necessary lead in promoting healthy and sustainable dietary guidelines for all.

Sustainable Healthy Diets are dietary patterns that promote all dimensions of individuals’ health and well-being; have low environmental pressure and impact; are accessible, affordable, safe and equitable; and are culturally acceptable. The aims of Sustainable Healthy Diets are to (a) promote optimal growth and development and support physical, mental, and social well-being at all life stages for present and future generations; (b) contribute to preventing all forms of malnutrition (i.e., undernutrition, micronutrient deficiency, overweight and obesity); (c) reduce the risk of diet-related NCDs; (d) support the preservation of biodiversity and planetary health^(24)^.

A study by Springmann *et al*.^(25)^ modelled the impact on people's health, as well as on the environment by replacing animal-source foods with plant-based ones and looked at outcomes such as the reduction of premature mortality, improvement of nutrient levels and reducing environmental impact. The results demonstrate that a predominantly plant-based diet has positive effects on environmental pressure, on potential nutrient deficits and could decrease diet-related mortality. There needs to be a strong consultation and discussion on the impact of reduced animal-sourced foods on the protein intake of subpopulations such as the elderly. The socio-economic status of countries was taken into consideration when analysing the results, and the modelling showed that only low-income countries would have difficulties fulfilling the nutrient intake recommendations when changing to an exclusively plant-based diet^(25)^.

Due to the high burden of NCDs throughout the life course, the education of primary health care providers as to the risk factors could be an effective way to support changing patient behaviour. Risk identification and individual consultation offered to patients are recommended by WHO as effective and cost-effective, especially when combining consultations on dietary and physical activity behaviour. The main challenges for implementing diet, physical activity and weight management into primary care are the poor health literacy, beliefs and attitudes, the lack of dedicated clinical guidelines and protocols for primary care, the lack of defined scope of practice, outdated knowledge, skills and competence, misalignment of payment mechanisms for these services and insufficient information technology infrastructure and tools^(26)^.

A longer life brings great opportunities. Yet the extent to which we as individuals and society can benefit from these extra years depends heavily on one key factor - ‘health’. Healthy ageing is defined as the process of developing and maintaining the functional ability that enables well-being in older age and can be achieved with a commitment to action, an alignment of health systems, the development of age-friendly environments and more research. Healthy ageing is a process that spans the entire life course and is relevant to everyone, not just to those who are presently free of disease. The intrinsic capacity of the individual (i.e., the combination of all the individual's physical and mental – including psychosocial – capacities), at any point in time is determined by many factors, including underlying physiological and psychological changes, health-related behaviours and the presence or absence of disease. These, in turn, are strongly influenced by the environments in which people have lived throughout their lives, which are also strongly influenced by personal characteristics such as gender and race^(4,27)^.

Increasing age coincides with many physiological changes that increase the risk of undernutrition, affecting up to 22 % of individuals, with subsequent physical and cognitive impairments, including reduced bone and muscle mass, increased frailty, diminished cognitive function and ability to care for oneself, and thus a higher risk of becoming dependent on care^(21)^. Sensory impairments (a decreased sense of taste and smell, for example), poor oral health, isolation, loneliness and depression – individually or in combination – all increase the risk of undernutrition in older age. Ageing is associated with changes in body composition; after the age of 60 years, there can be a progressive decrease in body weight that results mainly from a decrease in lean mass with an increase in fat mass. However, a stable body weight can mask age-related changes in body composition. Older people who do not consume enough protein are at increased risk of developing sarcopenia, a condition that leads to age-related loss of muscle mass and strength, osteoporosis and impaired immune response. Evidence indicates that oral nutritional supplements for older adults with undernutrition can significantly reduce mortality and improve body composition^(21)^.

Protein absorption decreases with age. Low protein intake is associated with loss of lean body mass, and standard protein intake, i.e., the daily recommended protein intake for adults, may not be sufficient for older people. The effects of exercise can be enhanced by combining it with increased protein intake and other nutritional interventions to prevent age-related loss of muscle mass and strength. Dietary counselling is important to ensure a healthy diet that provides adequate amounts of energy, protein and micronutrients and should be encouraged for all older people, including those who are at risk of or affected by undernutrition^(28)^.

Healthier populations are achieved through multi-sectoral actions that are not limited to health systems alone, though often using the stewardship, advocacy and regulatory functions of health ministries^(21)^. Countries vary widely in their ability to take action against NCDs. Progress has been limited, even though many recommendations exist. Political leadership and responsibility are needed at all levels from capitals to villages. Governments should identify and implement a specific set of priorities within the overall NCD and mental health agenda, based on public health needs, reorient health systems to include health promotion and the prevention and control of NCDs and mental health and set a whole-of-society approach to NCDs. Further, experiences and challenges including policy models that work and develop a new economic paradigm for funding action on NCDs and mental health should be shared^(18)^.

## Dietary interventions to promote healthier ageing

At an individual level, the ageing process is ‘plastic’. It can be accelerated by poor diet, lack of physical activity and exposure to hazardous environmental factors, including cigarette smoke and air pollution^(6)^. Conversely, ageing can be slowed through healthier lifestyles and by more favourable environments^(29)^. As a consequence, adopting healthier dietary patterns and avoiding obesity are important strategies to enable individuals to age more healthily and to minimise the risk of multiple age-related diseases^(30)^. The mechanisms through which altered diet improves ageing are not well understood, but it seems likely that a wide range of food-derived factors counteracts the molecular damage and mitigates the associated functional changes that are hallmarks of the ageing process^(13,14)^. Such damage is due to normal cellular processes, including inflammation, metabolic stress and oxidative stress/redox changes. Evidence that dietary exposures pre-pregnancy^(31)^ and during the first 1000 days of life^(32)^ influence the ageing trajectory supports the idea that better diets throughout the life course will maximise the potential for healthier ageing. However, improving eating behaviours in mid to later life is also likely to be beneficial^(30)^.

**Fig. 1.**
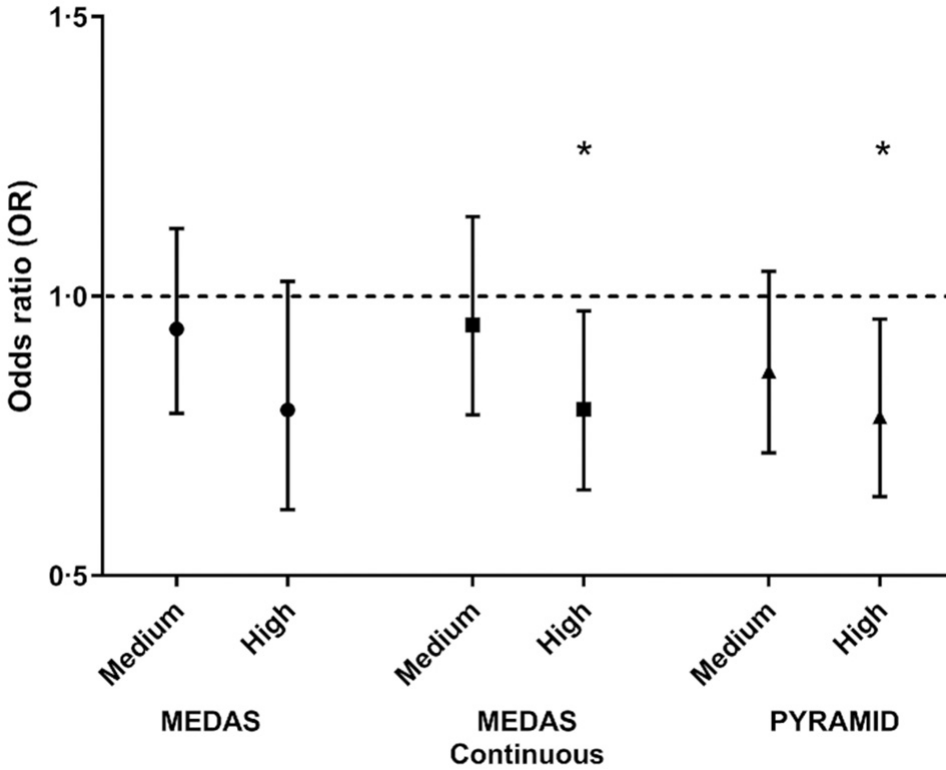
Associations between Mediterranean diet adherence and risk of poor cognitive performance assessed by the Hopkins Verbal Learning Test among participants (*n* 7589) in the European Prospective Investigation into Cancer and Nutrition-Norfolk study. Poor performance was defined as a score in the bottom 10 % of the population distribution of the test. Results are expressed as odds ratios (ORs) plus 95 % CIs for poor cognitive performance with medium and high compared with the lowest tertile of Mediterranean diet adherence (dashed line) quantified using three different methods of assessing adherence. These were: (a) the standard Mediterranean Diet Adherence Screener (MEDAS) (score); (b) the MEDAS Continuous that is similar to the standard MEDAS but with points allocated on a continuous basis (i.e., between 0 and 1) depending on closeness to the dietary target; (c) the Pyramid score, a 15-point scoring system proposed by the Mediterranean Diet Foundation^(37)^. Further details of these scoring methods are provided by Shannon *et al.*^(36)^. *Significantly lower risk of poor cognitive performance compared with the lowest tertile of Mediterranean diet adherence (*P* < 0⋅05).

In the EPIC-Norfolk prospective cohort study, those middle-aged individuals exhibiting more beneficial health behaviours at baseline had substantially reduced risk of mortality during the subsequent 14 years of follow-up^(33)^. These health behaviours included not smoking, being physically active, modest alcohol intake and better diet^(33)^. The importance of the overall diet is emphasised by studies of adherence to the Mediterranean diet (MD), a plant-based eating pattern that is characterised by frugality and moderation and associated with conviviality, culinary activities, physical activity and adequate rest^(34)^. In the EPIC Elderly study of >75 000 men and women, aged 60+ years who were initially healthy (no coronary heart disease, stroke or cancer at enrolment), greater adherence to the MD was associated with lower subsequent mortality^(35)^. This study was carried out across ten European countries and found no evidence of heterogeneity between countries, suggesting that diet, not geography, was the critical determinant of mortality risk^(35)^. In the EPIC-Norfolk cohort, greater adherence to the MD was associated with better cognitive function and lower risk of poor cognition in older UK adults^(36)^ (Fig. 1). These observations have been strengthened by systematic reviews and meta-analyses which provide consistent evidence that higher MD adherence is associated with lower later life mortality and lower deaths from, or incidence of, major age-related disease^(38)^. Brain tissue volume falls during ageing and the rate of brain atrophy is increased in those with, or at risk of developing dementia. Using magnetic resonance imaging (MRI) to quantify brain volume in a birth cohort of people who were studied when aged 73 years, Luciano *et al*.^(39)^ observed that those with higher MD adherence had significantly lower rates of brain atrophy over the following 3 years.

Given the pervasive effect of multiple dietary factors in mitigating the whole body dysfunction that characterises ageing^(13)^, it is unsurprising that implementing widespread dietary change, i.e., improving eating patterns, has provided some of the strongest evidence for beneficial effects of nutrition on ageing-related outcomes. Survivors of an initial cardiovascular event who were randomised to a MD in the Lyon Diet Heart Study (a secondary prevention trial) had significantly lower risk of a subsequent cardiovascular event^(40)^. In the PREDIMED Study, middle-aged men and women at increased CVD risk who were randomised to a MD pattern with supplemental extra virgin olive oil or nuts had significantly lower CVD than those randomised to the control diet (advice to reduce dietary fat)^(41)^.

Unhealthy lifestyles, characterised by inadequate physical activity and excess consumption of energy-rich foods, have led to a worldwide epidemic of obesity^(42)^. Obesity is associated with chronic systemic inflammation, which causes pervasive damage to macromolecules in all cell types, tissues and organs that, in turn, contribute to greater risk of multiple age-related diseases^(43)^. Using data from the Swedish Twin Registry, Xu *et al*.^(44)^ reported that adiposity in middle age (mean 43 years) was a strong predictor of subsequent risk of dementia with those who were obese in middle age having 2.5 times greater dementia risk than those with BMI 20–25. More generally, there is growing evidence that several aspects of the modern lifestyle may have adverse effects on longevity^(44)^.

The public health challenge is to develop, and deliver, interventions that result in healthier ageing in ways that are acceptable to the participants, cost-effective, sustainable and scalable. One of the impediments to research in this area is the absence of a reliable indicator of healthy ageing^(45^^)^. Given the pervasive nature of ageing, it seems likely that panels of measures will have greater utility than individual biomarkers^(46,47)^. For example, assessing function across a range of physiological, psychological and social domains, as encapsulated in the Healthy Ageing Phenotype, may be a useful outcome measure in intervention studies attempting to enhance healthy ageing^(48)^.

To date, most relevant intervention studies have focused on prevention of common age-related diseases, rather than attempting to improve healthy ageing *per se*. Present evidence suggests that improving lifestyle (including avoiding obesity, being more physically active and adoption of healthier eating patterns such as the MD) will play a central role in lowering dementia risk^(49)^. The FINGER trial tested the hypothesis that a multi-domain intervention (diet, exercise, cognitive training and vascular risk monitoring) would reduce the rate of cognitive decline in at risk, older people from the general population^(50)^. After 2 years, those randomised to the intervention had reduced rates of decline in processing speed and executive function^(50)^, providing evidence that such multi-domain interventions have potential to improve cognitive function during ageing and to reduce dementia risk.

**Fig. 2.**
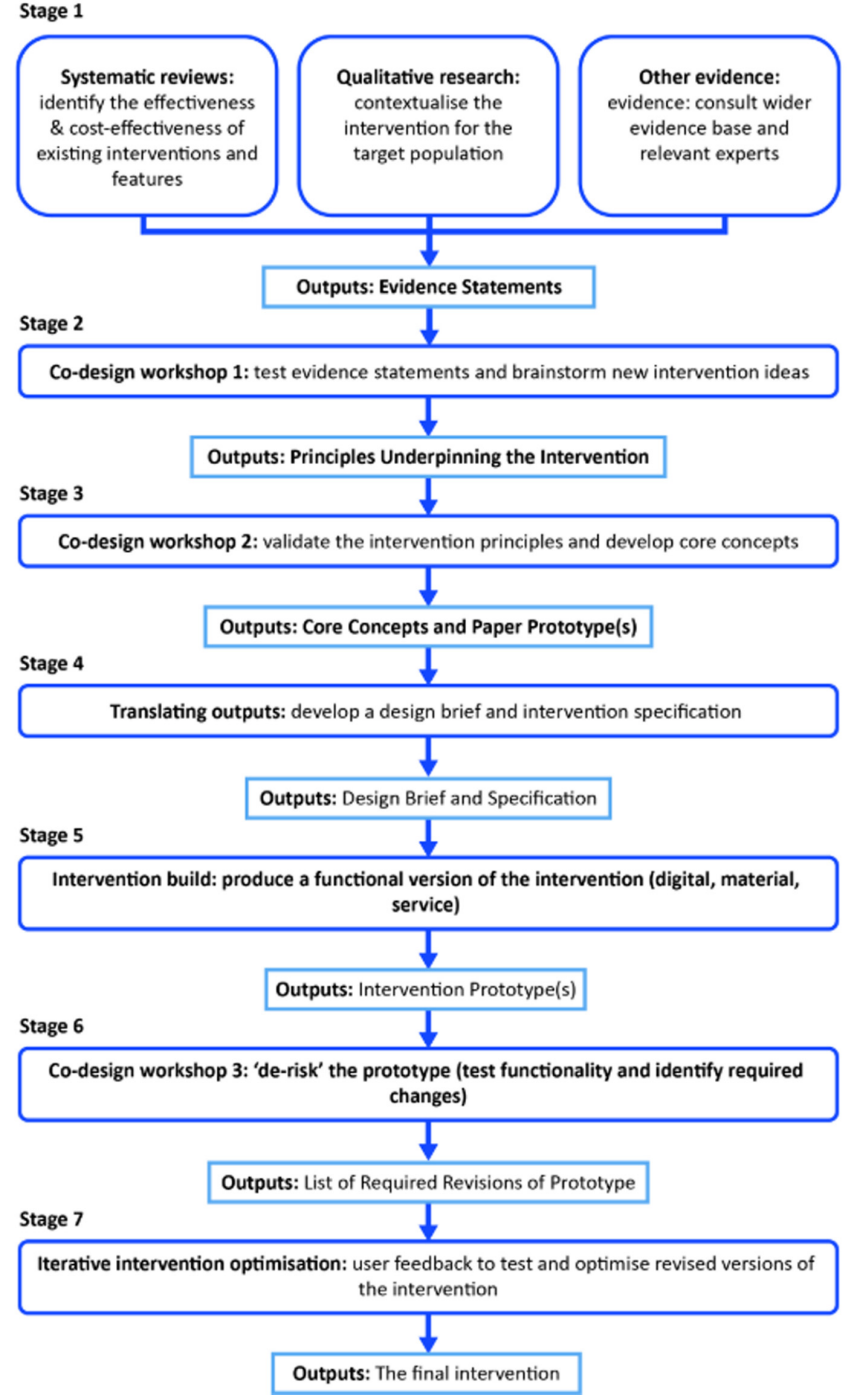
Overview of the systematic, sequential approach to intervention co-design and development used to develop the LEAP (Living, Eating, Activity and Planning) online platform. LEAP incorporates selected behaviour change techniques and is designed to provide personalised support in making changes to diet (to increase adherence to the Mediterranean dietary pattern), physical activity and social roles/ networks and to enhance healthy ageing^(53)^.

To be useful at a population level, interventions designed to improve lifestyle behaviours, and so enhance healthy ageing, should take account of the heterogeneity of older people (in terms of both needs and wants) and should be capable of implementation at scale, in a cost-effective manner. Personalised or stratified web-based interventions^(51,52)^ offer the potential to achieve these goals and to embed behaviour change techniques that improve effectiveness^(53)^ (Fig. 2). A pilot study of the web-based LEAP intervention which was designed to enhance healthy ageing by shifting eating patterns towards a MD, improving social networks and increasing physical activity^(54)^ was well-received by older people and showed good feasibility for larger studies^(54)^.

## Impact of (personalised) nutrition on healthy ageing from the perspective of phenotypic flexibility

From a metabolic point of view, optimal resilient ageing can be defined as the ability of a person to maintain or regain homeostasis in an ever-changing environment, especially in response to a wide range of stressors, i.e., a person's capacity to adapt. For a person to remain healthy in the presence of unpredictable changes require a high degree of flexibility or plasticity since the body needs to be able to adjust regulatory and adaptive processes, such as endocrine activity, metabolism and inflammatory signalling to control core metabolic biomarkers, such as plasma glucose, triacylglycerol and C-reactive protein^(55)^. Thus, a person who has optimal resilient ageing is, in fact, continuously changing their phenotype in response to an ever-changing environment, including the intake of food and/or levels of physical exercise. Their ability to adapt can act as an indicator for maintenance or improvement of physiological function or health status. The term ‘phenotypic flexibility’ or ‘resilience’ expresses the cumulative ability of overarching physiological processes (e.g., metabolism, inflammation and oxidation) to return to homeostatic levels after short-term perturbations^(56)^.

In the last decade, a new methodology has been established to quantify ‘phenotypic flexibility’ or ‘resilience’, for example, the analysis of post-prandial responses after a standardised mixed-meal challenge test provides a measure of metabolic resilience and allows the identification of dynamic phenotypic traits associated with early stages of NCDs^(57)^.

**Fig. 3.**
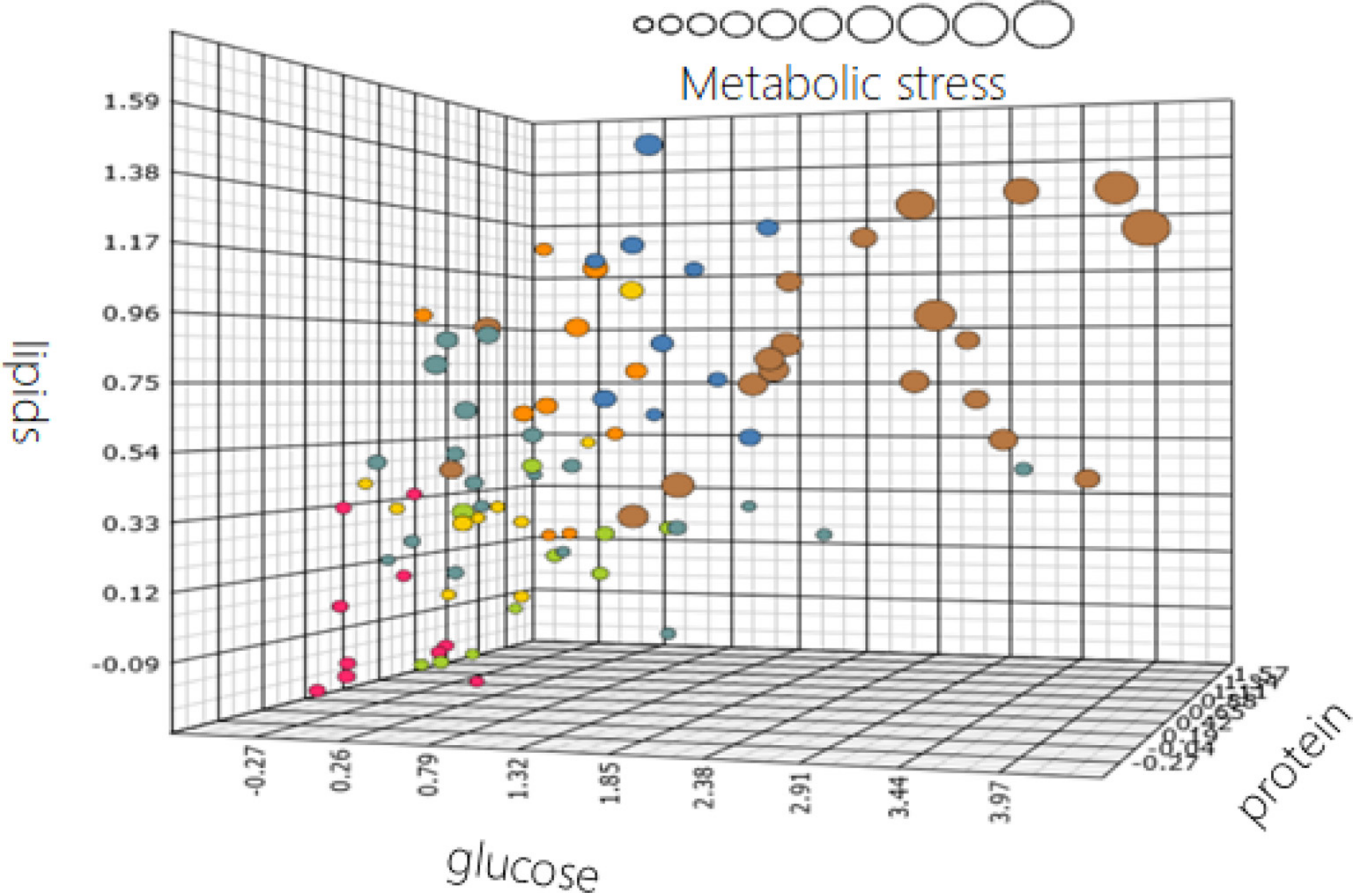
Health space that visualises the increase of a person's metabolic age with age, body fat and the presence of a metabolic disorder such as type II diabetes mellitus. The circles represent different male individuals from two studies focusing on health quantification by means of phenotypic flexibility^(64,65)^. The health space is defined by four axes that summarise all biomarkers related to the capacity to deal with ‘lipids or fats’, ‘glucose or carbohydrates’, ‘proteins’ and the size of the circle summarises the individual metabolic stress state referring to inflammation and oxidative stress. The colour coding of the circles have the following meaning: red, healthy young (age 20–30) and lean (low body fat percentage); marine blue, healthy elderly (age 60–70) and overweight (normal to high body fat percentage); green, healthy adults (age 30–60) with low body fat percentage; yellow and aqua-green, healthy adults (age 30–60) with normal body fat percentage; orange, healthy adults (age 30–60) with high body fat percentage; brown, male adults (age 30–60) with type II diabetes. Adapted from van den Broek *et al.*^(65)^.

This concept of phenotypic flexibility or challenge testing has been used in several nutritional intervention studies to quantify the health effect of a specific intervention on health including health modulation by nutrition^(55,58–63)^. However, there was a need for a generic holistic standardised challenge test that would be able to pick up the early signs of individual homeostatic disturbances as well as assessing (individual) health benefits from nutrition by showing an optimised response to the challenge test. Based on an extensive literature evaluation, the so-called PhenFlex challenge test was developed, which is a 500 ml drink containing 60 g of palm olein, 75 g of glucose and 20 g of casein^(57)^. The idea was that the collection of a multitude of biomarker response-profiles reflecting defined and accepted biological processes followed by sophisticated multivariate statistical analyses could be more powerful in detecting early changes than the limited set of individual biomarkers/single blood markers after an overnight fast. To investigate if this phenotypic flexibility approach had potential, a number of human volunteer studies have been performed in type II diabetics as well as in healthy populations^(64,65)^. It has been shown that quantification of phenotypic flexibility via the PhenFlex challenge test could discriminate between young, lean male and female persons as compared to overweight and obese elderly within the healthy range of the population^(65)^. It has also been shown that increasing body fat increases the so-called metabolic age of persons^(64,65)^. Furthermore, cardiometabolic disease, such as type II diabetes, even further increases the metabolic age of individuals^(65)^. Fig. 3 shows the visualisation of this decrease in optimal resilient ageing with increasing body fat and the presence of metabolic disease.

**Fig. 4.**
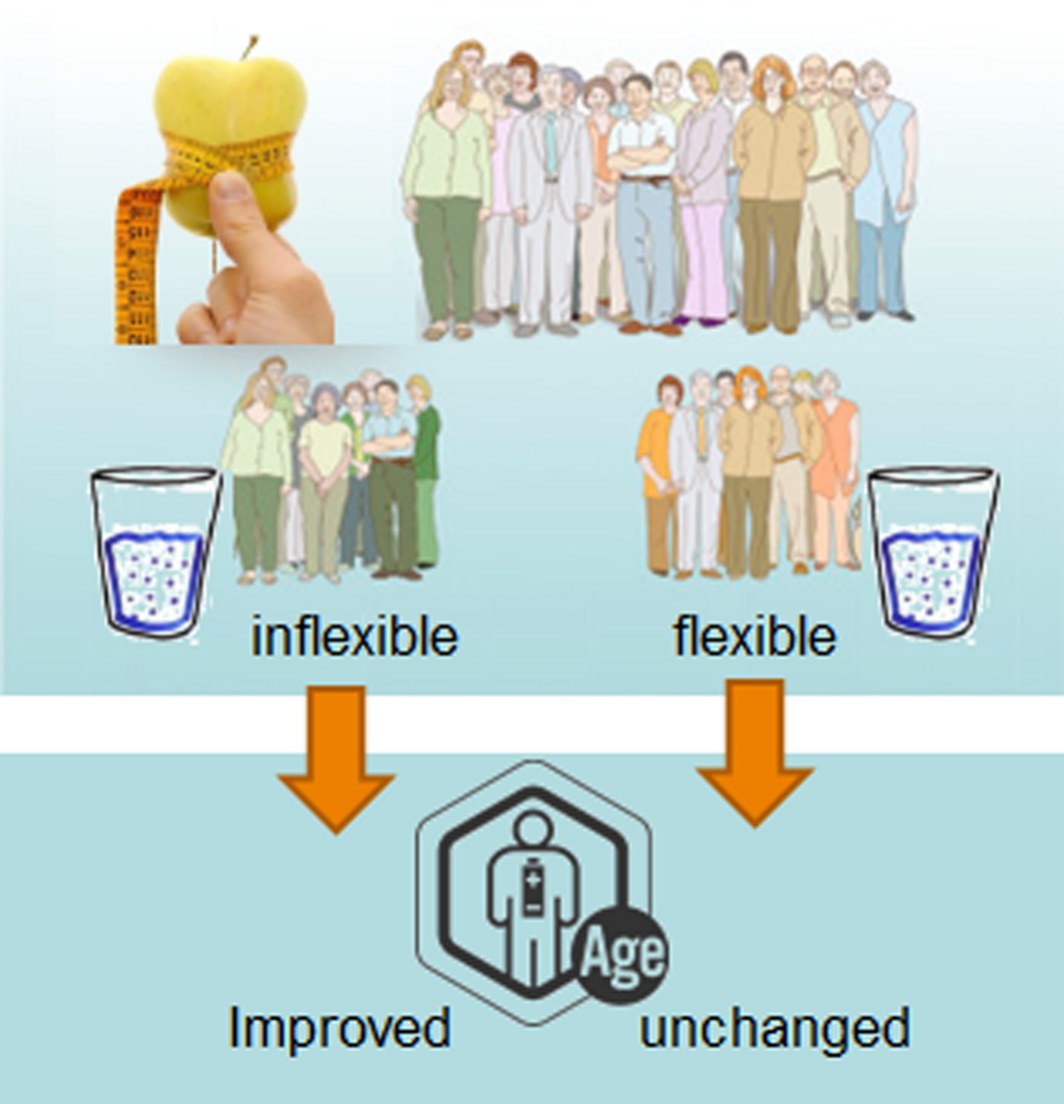
In a human intervention study, focusing on the effect of weight loss (20 % reduction in energy intake for 12 weeks) on ‘resilient ageing’, the quantification of phenotypic flexibility identified subjects with different susceptibility to weight loss-mediated metabolic improvements. Persons who had a reduced resilience (inflexible persons) showed a significant metabolic improvement after 12 weeks of intervention, whereas persons with normal resilience had an unaltered metabolic age.

Further, this standardised phenotypic flexibility toolbox demonstrated the health benefit of whole grain wheat on intrahepatic lipid, liver resilience, as well as inflammatory resilience, hypothetically by modulating the hepatic lipid efflux of obese male and female persons^(63,66)^. Another study, focusing on the health benefits of reduced caloric intake in overweight and obese persons showed that the quantification of phenotypic flexibility could discriminate between responders and non-responders to this lifestyle intervention. Persons who have relatively high flexibility at baseline (which translates to a relative young metabolic age) did not change their metabolic health state, although they lost on average 5 kg of weight over a period of 12 weeks (so-called non-responders), whereas persons with a relative high metabolic age at baseline improve their health as a result of the weight loss (so-called responders). Phenotypic measures showed improvement of several clinical markers, but also a significant reduction of intra-organ fat such as liver fat and intra-abdominal adipose tissue, which did not change in the non-responders, who already had significant lower intra-organ fat as compared to the responders (Fig. 4). This could also provide an explanation for why the mixed-meal challenge was not superior to measurements in the fasted state to assess metabolic health in a weight loss study in abdominally obese men^(67)^.

**Fig. 5.**
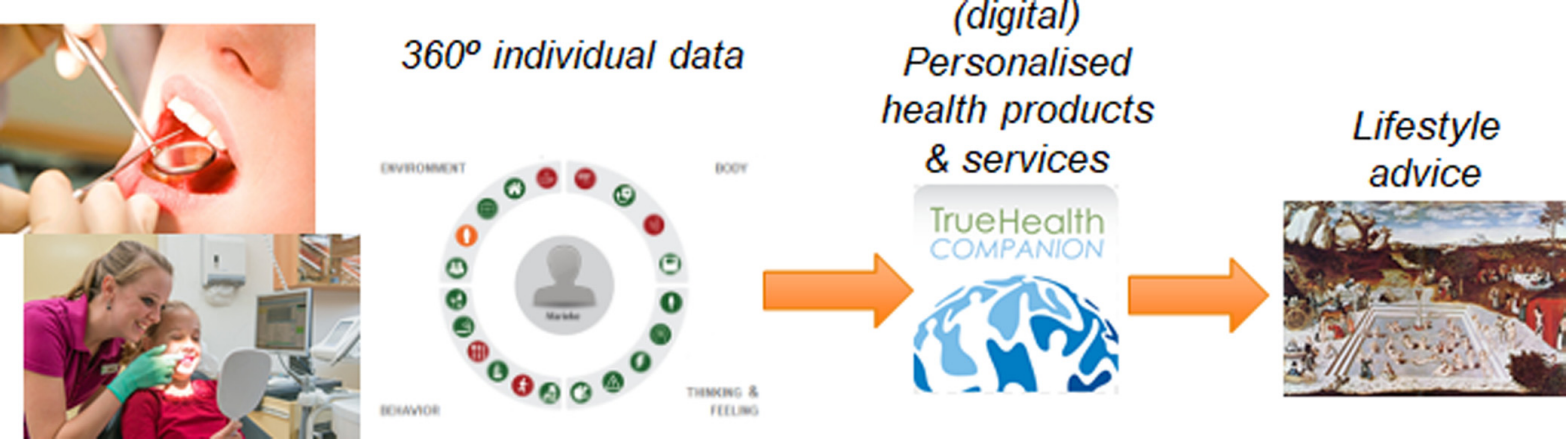
The envisioned transition from lifespan to healthspan: persons should have a regular health check through the lifespan like presently is the case within dentistry. Based on digital 360° individual data on a person's biology (including phenotypic flexibility), thinking and feeling, lifestyle behaviour and contextual environment, it can be determined if a person is following their optimal ageing trajectory. When deviation from this trajectory is noticed, personalized nutrition and lifestyle advice can be provided, thereby adding life to years by preventing non-communicable diseases (NCDs).

It is envisioned that nutrition in 10 years will be much more personal, i.e., the use of individual-specific information, founded in evidence-based science, to promote dietary behaviour change that may result in measurable health benefits^(68)^. The nutritional intervention or dietary advice will be based on a diagnosis, by using personal health data including biological (phenotypic flexibility) as well as behavioural measures. A systems-biology model will be used to translate the personal 360° health diagnosis into personalised nutritional advice. This model is tailored to specific personal preferences and goals, to gain better adherence to the diet^(3,69)^. When progressing from Lifespan to Healthspan, it is envisioned that personal health data collection will start at an early age. For example, from a young age, we go to the dentist twice a year in order to address and prevent oral problems. But for the greatest good – our health – we do not follow this type of prevention. Why not a regular routine health check for everyone? It is envisioned that such a health check could be based on an overarching view of health that focuses on a person's biology but is integrated with their thoughts and feelings, lifestyle behaviour and context^(68)^. By evaluating a person's health data over time, it may enable one to monitor a person's phenotypic flexibility, i.e., how a person is responding to stressors over a longer period of time. Based on derailments from a person's predicted health trajectory, notifications could be sent from a digital ‘health companion’, with advice on nutrition and lifestyle tailored to their biology and preferences. People could be guided to services and products to restore them to their resilient ageing trajectory. This could facilitate the transition from the present health care system that focuses on ‘disease treatment’ to a system that promotes and delivers real ‘health care’, i.e., a society focusing on the proactive maintenance of health and prevention of NCDs, thereby adding life to years (Fig. 5)!

## Conclusion

As discussed in the present paper, many countries are witnessing a marked increase in longevity and with this increased lifespan the concomitant desire for maximising healthspan. Unfortunately, greater life expectancy is often accompanied by more years of ill-health due to both physical and mental deterioration with its associated escalating costs for health and social care. Although good nutrition is fundamental for good health, the specific dietary interventions and/or nutrients that can enhance individual healthspan remain poorly understood. As discussed, there is growing evidence that (1) access to better nutrition; (2) improved immunity and response to disease/inflammation; (3) functioning senses (i.e., sight, taste and smell) and mobility; (4) the ability to maintain homeostasis or regain homeostasis in response to stress/stressors, may enhance how individuals age. This emphasises the need for innovative studies on dietary interventions to improve healthy ageing, especially utilising validated, and widely accepted, quantitative physiological biomarkers that measure key functions and are responsive to lifestyle-based interventions.
